# Identification of FDA-Approved Drugs as Potential Inhibitors of WEE2: Structure-Based Virtual Screening and Molecular Dynamics with Perspectives for Machine Learning-Assisted Prioritization

**DOI:** 10.3390/life16020185

**Published:** 2026-01-23

**Authors:** Shahid Ali, Abdelbaset Mohamed Elasbali, Wael Alzahrani, Taj Mohammad, Md. Imtaiyaz Hassan, Teng Zhou

**Affiliations:** 1 School of Cyberspace Security, Hainan University, Haikou 570228, China; ali.ali.md111@gmail.com; 2 Department of Clinical Laboratory Science, College of Applied Sciences-Qurayyat, Jouf University, Qurayyat 77451, Saudi Arabia; 3 Centre for Interdisciplinary Research in Basic Sciences, Jamia Millia Islamia, New Delhi 110025, India; taj144796@st.jmi.ac.in (T.M.);

**Keywords:** Wee1-like protein kinase 2, non-hormonal contraception, fertility modulation, drug repurposing, virtual screening, machine learning

## Abstract

Wee1-like protein kinase 2 (WEE2) is an oocyte-specific kinase that regulates meiotic arrest and fertilization. Its largely restricted expression in female germ cells and absence in somatic tissues make it a highly selective target for reproductive health interventions. Despite its central role in human fertility, no clinically approved WEE2 modulator is available. In this study, we employed an integrated in silico approach that combines structure-based virtual screening, molecular dynamics (MD) simulations, and MM-PBSA free-energy calculations to identify repurposed drug candidates with potential WEE2 inhibitory activity. Screening of ~3800 DrugBank compounds against the WEE2 catalytic domain yielded ten high-affinity hits, from which Midostaurin and Nilotinib emerged as the most mechanistically relevant based on kinase-targeting properties and pharmacological profiles. Docking analyses revealed strong binding affinities (−11.5 and −11.3 kcal/mol) and interaction fingerprints highly similar to the reference inhibitor MK1775, including key contacts with hinge-region residues Val220, Tyr291, and Cys292. All-atom MD simulations for 300 ns demonstrated that both compounds induce stable protein–ligand complexes with minimal conformational drift, decreased residual flexibility, preserved compactness, and stable intramolecular hydrogen-bond networks. Principal component and free-energy landscape analyses further indicate restricted conformational sampling of WEE2 upon ligand binding, supporting ligand-induced stabilization of the catalytic domain. MM-PBSA calculations confirmed favorable binding free energies for Midostaurin (−18.78 ± 2.23 kJ/mol) and Nilotinib (−17.47 ± 2.95 kJ/mol), exceeding that of MK1775. To increase the translational prioritization of candidate hits, we place our structure-based pipeline in the context of modern machine learning (ML) and deep learning (DL)-enabled virtual screening workflows. ML/DL rescoring and graph-based molecular property predictors can rapidly re-rank docking hits and estimate absorption, distribution, metabolism, excretion, and toxicity (ADMET) liabilities before in vitro evaluation.

## 1. Introduction

Protein kinases are central regulators of intracellular signaling, controlling processes such as cell division, differentiation, apoptosis, and metabolism [1]. Dysregulation of kinase activity is implicated in a wide spectrum of diseases, including cancer, neurological disorders, and reproductive pathologies [2,3]. Within this superfamily, the Wee kinase family plays a key role in regulating cell cycle checkpoints through the inhibitory phosphorylation of cyclin-dependent kinase 1 (CDK1) [4]. This family includes Wee1, WEE2 (also known as Wee1B), and PKMYT1 [5]. Although Wee1 and PKMYT1 are somatically expressed protein kinases with well-studied roles in DNA damage repair and mitotic function, WEE2 is an oocyte-specific kinase that is mainly expressed in the female germline [6]. Importantly, the absence of WEE2 expression in somatic tissues makes it a highly selective target with minimal risk of off-target effects, positioning it as an especially attractive candidate for reproductive health interventions [7].

During oocyte maturation, WEE2 is critical in supporting meiotic arrest [8]. Meiosis in mammalian oocytes is arrested at prophase I for a long time until the luteinizing hormone surge induces resumption of meiotic progression [9]. This arrest is sustained by WEE2, which phosphorylates CDK1 at tyrosine 15, thus preventing activation of the maturation-promoting factor (MPF; CDK1–cyclin B complex) [5]. WEE2 function is also critical for the release from metaphase II during fertilization, allowing for pronuclear fusion and normal development to the zygote stage [10]. WEE2 mutations have been linked to total fertilization failure in women undergoing assisted reproductive techniques, particularly intracytoplasmic sperm injection (ICSI) [11]. Microinjection of WEE2 complementary RNA into oocytes that were unable to be fertilized has restored fertilization and highlighted the importance of this specific kinase in human fertility [12].

WEE2 serves the dual function of both maintaining meiotic arrest and enhancing fertilization and represents a unique target for therapeutic intervention in reproductive medicine [7]. This inhibition of WEE2, on one hand, presents a novel non-hormonal contraceptive approach. Unlike hormonal contraceptives, which act systemically and are often associated with side effects such as metabolic disturbances, mood alterations, and increased cardiovascular risk, a WEE2-targeted approach would act locally at the oocyte to block fertilization while leaving systemic endocrine physiology largely unaffected [7]. Therefore, WEE2 inhibition represents a compelling option for women who desire a safe, effective, reversible, and non-hormonal form of contraception. Conversely, restoring or enhancing WEE2 activity could represent an innovative therapeutic strategy for infertility cases linked to WEE2 dysfunction, offering potential benefits on both sides of the reproductive spectrum—contraception and fertility treatment [13].

This line of research has opened up therapeutic opportunities that can be explored by a drug-repurposing strategy [14]. Drug repurposing is when new indications are discovered for drugs that have already been approved for human use, or are close to clinical development [15]. Since the safety, pharmacokinetic, and toxicological profiles of these compounds are already known, repurposing has the potential to drastically reduce the time and cost of drug development [16]. Repurposing provides an earlier route towards identifying such candidate molecules with clinical translation potential in cases like WEE2, where selective inhibitors or activators have not yet been developed for clinical use. High-throughput screening of large libraries of compounds in the life sciences is an experimental pipeline that is expensive, slow, and features low yield rates in drug discovery. However, experimental methods have limitations, and structure-based drug discovery has emerged as a powerful adjunct to experimental methods in computational biology, molecular modeling, and bioinformatics with recent advances [17]. Structure-based approaches use 3D protein structures (obtained through X-ray crystallography or cryo-EM) to predict and assess protein–small-molecule binding [18]. It enables the rational prioritization of compounds before expensive laboratory testing.

Virtual screening has become an essential computational approach in the early stages of drug discovery [19]. Virtual screening involves evaluating thousands of compounds against a defined protein target to identify those with favorable binding characteristics [20]. Molecular docking is an integral part of this process, which helps to predict the orientation, binding mode, and binding energy of small molecules in the active site of the target [21]. Docking results can yield valuable details about potential lead compounds, such as their ability to form hydrogen bonds, hydrophobic contacts, and other stabilizing contacts within the binding pocket. Docking is useful to form initial hypotheses; however, it is a static approach and does not incorporate the essential dynamic aspects of protein–ligand interactions. That is the reason why molecular dynamics (MD) simulations are frequently used as a complementary tool [22]. MD simulations offer time-resolved views on protein–ligand complexes in a near-physiological and solvated environment [23]. It enables probing of binding affinity, conformational plasticity, and crucial inter-monomer interactions on nanosecond-to-microsecond timescales [24]. The integration of docking and MD simulations provides more details about binding events, therefore increasing the confidence of computational predictions [25].

Collectively, these computational approaches can speed up the identification of selective WEE2 modulators. Therapeutics are often discovered through conventional drug-discovery methods that rely on screening large experimental libraries, a modern approach that is slow and expensive. Computational biology innovations allow these standard approaches to be complemented by more rapid in silico strategies. However, structure-based drug discovery, fueled by the advances of molecular modeling and bioinformatics, has proven to be a good approach to speed up lead compound identification [26]. Prioritization of potential drug candidates often involves rapid evaluation of hundreds of thousands of unique compounds in large chemical libraries, where the value of virtual screening lies. While molecular docking enables the estimation of the binding affinities and poses of small molecules in the binding pockets of proteins, the MD simulations reveal the dynamic stability and conformational adjustability of protein–ligand complexes [19].

Machine learning (ML) and deep learning (DL) have become increasingly integrated into structure-based virtual screening pipelines [27]. ML-based scoring functions and deep neural networks can re-rank docking poses, predict absorption, distribution, metabolism, excretion, and toxicity (ADMET) liabilities, and estimate off-target risks more rapidly than traditional computational methods [28]. These approaches reduce false positives and accelerate candidate prioritization before in vitro testing. Recent AI-driven tools, such as convolutional scoring models and graph-based predictors, offer reproducible strategies to complement docking and MD results. These approaches both complement and accelerate the structure-based methods used here and help prioritize candidates for biochemical validation [29]. We position our structure-based screening and MD simulations as a rigorously evaluated candidate-generation step that is readily amenable to ML/DL-based rescoring and property prediction. We propose a concise ML/DL-assisted prioritization follow-up that can be applied to our top docking hits to refine candidate ranking and to estimate likely ADMET profiles before bench validation. Recent toolkits and methodological advances offer practical, reproducible methods for performing such rescoring. In healthcare and drug discovery, AI has been applied to diagnostic imaging, disease prediction, and large-scale molecular screening [30]. Studies such as the use of self-organizing maps for MRI cancer-zone detection and XGBoost-based models for type 2 diabetes prediction illustrate the power of ML in biomedical decision-making [31]. Integrating such AI methodologies into drug-repurposing workflows can enhance candidate prioritization and reduce experimental burden.

Here, we employed an integrated molecular docking and MD simulation-based approach to identify potential WEE2 inhibitors from a library of repurposed drugs. A pragmatic approach to overcoming these challenges is to screen repurposed drugs that are known for their safety and efficacy, thereby reducing time and cost, given existing pharmacological knowledge. It enables the identification of new WEE2 modulators, as well as the characterization of their binding profiles and the structural determinants of selectivity. Ultimately, these studies can aid the design of further experimental assays, help prioritize candidates for additional refinement, and target non-hormonal contraception and fertility modification therapies.

## 2. Materials and Methods

### 2.1. Computational Tools and Web Resources

Virtual screening was carried out using drug-likeness evaluation, molecular docking, and MD simulations on an HP Z420 workstation. The 3D structure of the WEE2 protein was downloaded from the RCSB Protein Data Bank [PDB ID: 5VDK], and an FDA-approved and investigational compound library comprising around 3800 compounds in processed 3D formats was obtained from the DrugBank database [32]. Molecular docking was performed with InstaDock v1.2 [33] to model binding orientations and affinities of candidate molecules within the active site of WEE2. The protein–ligand complex was inspected and visualized using PyMOL (v3.1) [34] and Discovery Studio Visualizer (v2023) [35] software to observe binding poses, hydrogen bonding, hydrophobic interactions, and other spatial relationships. To evaluate dynamic stability and conformational changes under simulated physiological conditions, all-atom MD simulations were performed on the docked complexes using GROMACS (version 2022) [36]. These simulations were used to examine the retention of key interactions and the flexibility of the WEE2 backbone within the ligand–protein complexes.

### 2.2. Structure-Based Molecular Docking Screening

To identify potential WEE2 inhibitors, we performed structure-based molecular docking using the experimentally determined 3D structure of human WEE2 (PDB ID: 5VDK). Before docking, the protein structure was prepared by removing water molecules and co-crystallized ligands to ensure a clean binding environment. A blind docking grid was defined, centered at coordinates (−13.229, −17.901, −5.688) with dimensions of 75 × 74 × 58 Å, covering the entire protein to allow unbiased exploration of potential binding sites. This setup permitted the investigation of putative binding regions without targeting any binding site. Ligand structures from DrugBank were pre-processed to ensure correct protonation states and 3D geometries. Docking was carried out with InstaDock v1.2, which evaluates possible ligand orientations and calculates binding affinities, where more negative docking scores indicate higher binding potential. The docking workflow was implemented using the InstaDock protocol because of its validated scoring function, suitability for kinase targets, and efficient handling of large compound libraries. InstaDock employs a hybrid search algorithm combining Lamarckian genetic optimization and gradient-based local refinement, enabling reliable generation of energetically favorable binding poses. We selected InstaDock because it consistently reproduces crystallographic binding configurations of kinase inhibitors, including the co-crystallized WEE2 inhibitor MK1775, thereby enhancing confidence in pose accuracy and affinity ranking (Supplementary Figure S1). The resulting protein–ligand complexes were visualized in PyMOL and Discovery Studio Visualizer to analyze binding poses, hydrogen bonds, hydrophobic contacts, and π-π interactions. A list of compounds displaying relevant molecular interactions and stable binding conformations was further considered for downstream analysis.

### 2.3. Drug Profiling and Prioritization

To assess the translational potential of the hits identified, a comprehensive drug profiling analysis was performed on the shortlisted FDA-approved compounds obtained from molecular docking-based virtual screening against WEE2. Pharmacological data for each compound (primary molecular targets, mechanism of action, and approved therapeutic indications) were retrieved from publicly available databases, including DrugBank [32], PubChem [37], and ChEMBL [38]. Clinical relevance, therapeutic categories, and currently reported kinase activity were then abstracted from the literature. The profiling was intended to evaluate the appropriateness of each compound for possible repurposing against WEE2, emphasizing drugs with previously established activity against kinases, cell cycle regulators, or reproductive health pathways. We compared compounds based on (i) overlap of their pharmacological mechanism with WEE2 kinase biology, (ii) therapeutic relevance to fertility modulation or of reproductive system regulation, and (iii) safety and clinical applicability. Additionally, an AI-assisted prioritization step using an OpenAI GPT-based large language model (LLM) was applied to contextualize pharmacological information and highlight literature-supported kinase associations. This LLM-driven reasoning complements conventional profiling and reflects a modern, machine learning-assisted drug screening approach. The GPT-based LLM was used exclusively to assist with literature organization and contextual interpretation of pharmacological data. No predictive, analytical, or numerical outputs were generated by the model. All final data, rankings, and mechanistic interpretations were manually verified using primary scientific sources.

### 2.4. Molecular Dynamics Simulations

All-atom MD simulations were performed to assess the dynamic stability and behavior of unbound WEE2 and its ligand-bound complexes predicted from molecular docking. Docked poses of the compounds, the reference inhibitor MK1775, and unbound WEE2 protein were used as the starting structures. For each system, independent 300 ns simulations were performed using the GROMOS 54A7 force field [39] implemented in the GROMACS 2022 software package. The ligand topologies were prepared using the PRODRG server, which is adjusted in accordance with the force field for use [40]. Cubic periodic boxes containing SPC216 [41] molecules were used to solvate the protein–ligand systems, which were spaced 10 Å for an edge buffer around the solute. Electroneutrality was achieved by adding counterions (12 Na^+^). After setting up the system geometry, the steepest descent energy minimization was carried out for 1500 steps to generate an orientation with minimal steric clashes and minimized atomic positions. The resulting minimized systems were then equilibrated into an NVT ensemble (constant number of particles, volume, and temperature) for 1 ns, followed by an NPT ensemble (constant number of particles, pressure, and temperature) for another 1 ns. Following this, production MD simulations of WEE2–ligand complexes for 300 ns were performed using the NPT ensemble to characterize their structural and dynamic features. The simulations were performed at 300 K using the V-rescale thermostat and maintained at 1 bar using the Parrinello–Rahman barostat. Energy minimization was performed until forces fell below 1000 kJ/mol·nm. A 2 fs timestep was used with LINCS constraints on hydrogen bonds. Long-range electrostatics were computed with PME, and van der Waals interactions used a 1.0 nm cutoff. VMD [42] was used to visualize the trajectory, to analyze the interaction, and QtGrace was used to prepare the quantitative evaluation and create plots of the simulation results.

### 2.5. Principal Component Analysis

To further reveal the major motions and conformational landscape of WEE2 free from ligands and in complex with the final compounds, principal component analysis (PCA) was performed. It helps to determine the overall motions of atoms from MD trajectories, thus providing additional information about the structural stability and conformational flexibilities of the systems [43]. The trajectories were initially processed to extract the coordinates of the alpha-carbon (*C*_α_) atoms, which capture the essential protein backbone dynamics. The matrix describing the fluctuations of the atomic positions was then generated, diagonalized to obtain eigenvectors and eigenvalues, which correspond, respectively, to the principal modes of motion and their relative contributions. The most structural changes within protein–ligand complexes were characterized by concentrating on the first few principal components (PCs) that accounted for the majority of variance. This provided a global view of how the binding of ligands modulates the degrees of freedom of WEE2 with respect to its unbound form, emphasizing potential functional implications of the interactions.

### 2.6. Free-Energy Landscape Analysis

To investigate the structural stability and conformational transitions of the WEE2 in both free and complex states with the prioritized ligands, free-energy landscape (FEL) analysis was performed. This is a useful methodology that allows for the gathering of information about the conformational states sampled throughout the simulation and the associated relative energy barriers between these states [44]. The FELs were built out of the MD trajectories of all systems by reducing the number of dimensions of the principal motions aligned along the first two eigenvectors (EV1 and EV2) generated from the principal component analysis. The dominant collective motions of a system are captured as eigenvectors, which allow the conformational space to be represented in lower dimensionality. Subsequently, the free-energy surfaces were obtained by mapping the Gibbs free energy as a function of these principal components, where lower-energy regions correspond to more stable conformations and higher-energy regions indicate fewer favorable states. This allowed for a grouping of distinct basins and potential metastable states, providing insight into the conformational sampling and energy barriers related to the behavior of WEE2 alone, and in complex with the putative inhibitors. This gave us a basis for comparison to measure how the impact of ligand binding affects the dynamic landscape and stability of the protein.

### 2.7. MM-PBSA Calculations

Binding free energies of the WEE2–ligand complexes were calculated using the molecular mechanics Poisson–Boltzmann surface area (MM-PBSA) approach [45]. This method strikes a balance between computational efficiency and robustness at predicting interaction energies. The *g_mmpbsa* package was used for MM-PBSA calculations in combination with GROMACS [46]. From the equilibrated simulation trajectory, 50 ns of the equilibrium trajectory was analyzed, and the structural coordinate frames were extracted at 10 ps intervals to adequately sample the conformational space. These calculations included contributions from molecular mechanics energies, polar solvation energy calculated by the Poisson–Boltzmann model, and non-polar solvation energy predicted based on solvent-accessible surface area. Solvation electrostatic potentials were calculated with the Adaptive Poisson–Boltzmann Solver (APBS). The binding free energy (Δ*G*_binding_) was calculated as

∆*G*_binding_ = *G*_complex_ − (*G*_receptor_ + *G*_ligand_)

where *G*_complex_ represents the total free energy of the protein–ligand complex, while *G*_protein_ and *G*_ligand_ correspond to the free energies of the isolated protein and ligand, respectively.

## 3. Results and Discussion

### 3.1. Molecular Docking Screening

The original compound library was derived from the DrugBank database and included about 3800 repurposed drugs with varied chemical scaffolds and biological activities. Biologics and non-drug-like entities were filtered out from this dataset to yield a collection of ~3600 small-molecule compounds to be used for receptor-based virtual screening while minimizing false-positive hits. To evaluate the binding affinity and the action potential, each of the above filtered compounds was docked individually at the WEE2 active site. Docking scores for a subset of these compounds demonstrate appreciable binding, ranging from −10.6 to −12.6 kcal/mol. These compounds also exhibited better binding than the reference WEE2 inhibitor MK1775 (Table 1); then, the top ten compounds with the most favorable binding affinities were selected from this subset. These results highlight virtual screening as a valuable resource for identifying effective WEE2 inhibitors and may support their use in non-hormonal contraceptives and fertility-modifying agents.

### 3.2. Drug Profiling and Prioritization

Drug profiles of the screened compounds from molecular docking were subsequently analyzed. While many of the FDA-approved drugs we identified have strong binding affinity for WEE2, we selected the candidates based on their therapeutic profiles and mechanistic relevance to kinase biology. From the shortlisted compounds, Midostaurin and Nilotinib were identified as the most appropriate candidates for further downstream evaluation (Table 2). Midostaurin is a spectrum multi-kinase inhibitor that is clinically approved for acute myeloid leukemia and advanced systemic mastocytosis [47]. Its known activity against serine/threonine and tyrosine kinases provides a strong rationale for its potential repurposing against WEE2 [48]. Likewise, Nilotinib is a second-generation tyrosine kinase inhibitor that is commonly used for chronic myeloid leukemia management [49]. AI-assisted evaluation further supported these selections by contextualizing pharmacological and literature-based evidence. This complementary machine learning-assisted reasoning reinforced the prioritization of Midostaurin and Nilotinib as promising candidates for WEE2-targeted, non-hormonal contraceptive development. Due to its high specificity for ATP-binding pockets and satisfactory long-term clinical safety profile, Nilotinib is a promising candidate for repurposing as a WEE2 inhibitor. Based on the criteria we generated, the use of Midostaurin and Nilotinib can be explored as a WEE2-targeting anti-kinase-based strategy for the development of non-hormonal contraceptive and fertility-regulating pharmacological agents. The combination of these two kinase inhibitors offers the greatest translational potential and should be validated preclinically and experimentally.

Because Midostaurin and Nilotinib are multi-kinase inhibitors, we also examined their potential off-target risks. Literature reports describe broad kinase inhibition spectra, some associated with hematologic and cardiovascular adverse effects. To address this concern, we performed additional docking against WEE1, the closest somatic homolog of WEE2. Docking against a WEE1 structural model predicted marginally weaker affinity for both compounds (Midostaurin −10.1 kcal/mol; Nilotinib −9.9 kcal/mol), suggesting a possible preference for WEE2. However, docking alone cannot establish selectivity—biochemical WEE2/WEE1 profiling is required to confirm these computational predictions.

### 3.3. Interaction Study

**Figure 1:**
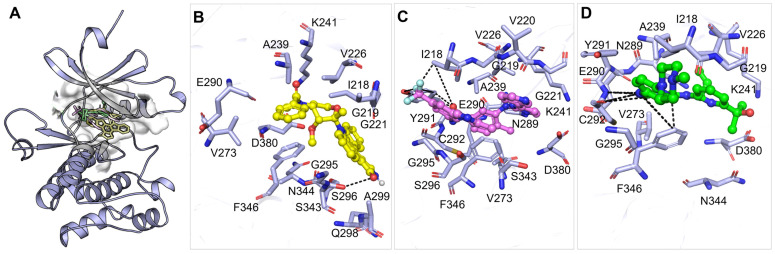
Binding interactions of WEE2 with Midostaurin, Nilotinib, and MK1775. (**A**) Overall view of WEE2 bound to each ligand. (**B**–**D**) Close-up images of the active-site binding pocket showing key interactions with Midostaurin (yellow), Nilotinib (magenta), and MK1775 (green), respectively.

This information is critical to ensure the specificity and potency of candidate inhibitors when selecting compounds for drug repurposing, while also helping to identify how to avoid nonspecific binding. WEE2 inhibitors whose structures were co-crystallized have provided insights into inactive conformation; crucial residues in the ATP-binding pocket (e.g., Ile218, Gly219, Val220, Glu221, Glu222, Val226, and Lys241) typically participate in polar interactions, which are key for the recognition of this class of inhibitors [50]. We used PyMOL and Discovery Studio Visualizer to carry out interaction profiling of Midostaurin and Nilotinib on WEE2. Both compounds were found to interact with important catalytic residues, including Asn344 and Asp380 in the Mg^2+^-binding region of the kinase (Figure 1). All three compounds are bound in the ATP-binding pocket of WEE2 and superimposed on each other (Figure 1A). Midostaurin and Nilotinib formed several favorable contacts within the WEE2 active pocket (Figure 1B,C). Notably, their binding modes were quite similar to the co-crystallized reference inhibitor MK1775, further supporting their promise as selective WEE2 inhibitors (Figure 1D).

**Figure 2:**
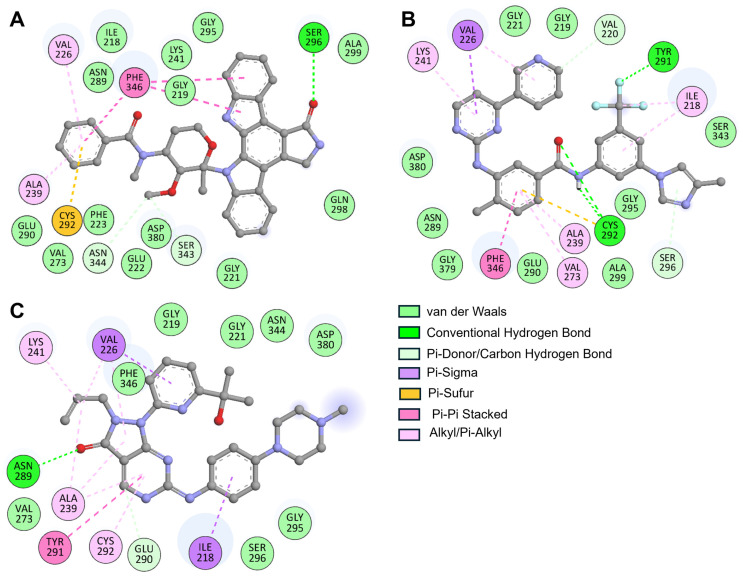
Two-dimensional interaction maps of WEE2–ligand complexes. Hydrogen bonds, hydrophobic contacts, and other key interactions are illustrated for (**A**) Midostaurin, (**B**) Nilotinib, and (**C**) MK1775.

We compared the binding profiles of the interactors with WEE2 to understand the different interaction patterns of Midostaurin and Nilotinib with WEE2 (Figure 2). In a more detailed analysis, we identified that Midostaurin formed three hydrogen bonds with the residues of the ATP-binding site, including Ser296, Ser243, and Ser244 (Figure 2A). In addition to these hydrogen bonds, Midostaurin also engaged in multiple hydrophobic contacts, van der Waals forces, and π–π stacking interactions with neighboring residues in the ATP-binding pocket, further reinforcing its stable and versatile binding mode with WEE2. In comparison, Nilotinib formed conventional hydrogen bonds with key residues Val220, Tyr291, Cys292, and Ser296, establishing a strong interaction network within the ATP-binding site. Beyond hydrogen bonding, it also engaged in hydrophobic contact and π–π interactions with surrounding residues, contributing to a stable and well-defined binding conformation in WEE2 (Figure 2B). Both Midostaurin and Nilotinib were found to have multiple common interacting residues with the reference inhibitor MK1775 based on a comparative analysis, all of which indicated common regions of binding within the WEE2 pocket (Figure 2C). Notably, both compounds were present in the active site region of WEE2, suggesting we could inhibit the functional activity of WEE2 like MK1775. Although docking and MD simulations cannot prove enzymatic inhibition, the observed binding patterns provide a strong structural rationale for a plausible inhibitory mechanism. Midostaurin and Nilotinib both occupy the ATP-binding cleft and reproduce key hinge-region interactions with Val220, Tyr291, and Cys292, residues that are also engaged by the reference inhibitor MK1775. This shared interaction fingerprint suggests a likely ATP-competitive mode of action. However, we acknowledge that computational binding alone does not confirm functional inhibition and therefore recommend experimental validation through in vitro WEE2 kinase assays, ATP-competition studies, and WEE2–WEE1 selectivity profiling. Overall, these results illustrate the broad spectrum of binding interactions of Midostaurin and Nilotinib and provide further support for both agents as candidates for experimental testing as WEE2-targeted non-hormonal contraceptives and fertility regulators.

### 3.4. MD Simulations

MD simulations require equilibration and stability of the system to ensure proper characterization of protein–ligand complexes [51]. The stability of WEE2, WEE2–Midostaurin, WEE2–Nilotinib, and WEE2-MK1775 complexes was calculated using the average values of the root mean square deviation (RMSD), root mean square fluctuation (RMSF), radius of gyration (*R*_g_), and solvent-accessible surface area (SASA) over the trajectory for the four systems and presented in Table 3. The results showed only small changes in the conformational dynamics and stability of WEE2 before and after ligand binding during the simulation time, as discussed in the ensuing sections.

#### 3.4.1. Structural Dynamics and Residual Vibrations

**Figure 3:**
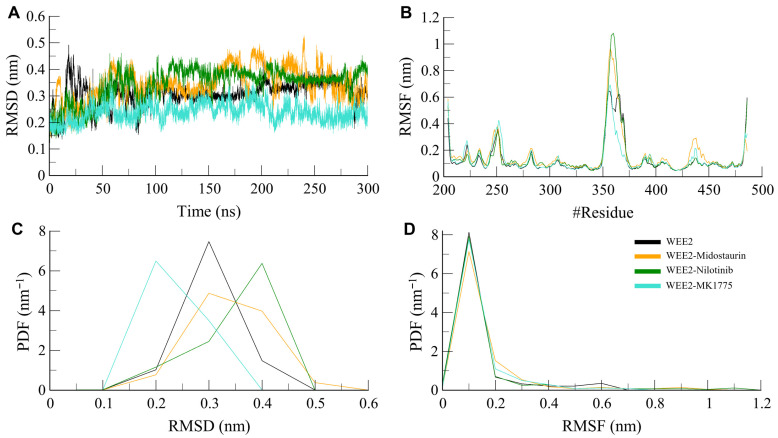
Structural stability of WEE2 and its ligand complexes during MD simulations. (**A**) RMSD plots showing conformational stability of free and ligand-bound WEE2. (**B**) RMSF plots depicting residue-wise flexibility. (**C**,**D**) The lower panels show the corresponding probability density functions (PDFs) for RMSD and RMSF, respectively.

We analyzed the econometric aspects imposed on the structural flexibility of WEE2 alone as well as in complex with the selected ligands in terms of RMSD and RMSF for the entire simulation period. Table 3 reveals low average RMSD values of 0.31 nm, 0.34 nm, 0.34 nm, and 0.24 nm formed by WEE2, WEE2–Midostaurin, WEE2–Nilotinib, and WEE2-MK1775, respectively. RMSD plots indicated that all systems came to equilibrium in the first 50 ns of simulation, and that ligand binding induced some minor conformational fluctuations compared to free WEE2 (Figure 3A). Notably, WEE2-MK1775 exhibited the lowest RMSD, indicating increased structural condensation and stabilization. This observation was confirmed by probability density function (PDF) analysis, which showed that the RMSD values in the complexes had a higher probability of being clustered around ~0.2–0.4 nm as compared to (Figure 3C).

RMSF analysis revealed that, particularly in binding-site regions, residues showed lesser fluctuations in ligand-bound systems than for free WEE2 (Figure 3B). The RMSF average values for WEE2, WEE2–Midostaurin, WEE2–Nilotinib, and WEE2-MK1775 were 0.13 nm, 0.16 nm, 0.15 nm, and 0.13 nm, respectively. These values indicate that ligand binding generally limits local flexibility and contributes to structural stabilization of WEE2. Among both the elucidated ligands, Nilotinib produced the most pronounced decrease in fluctuations, further illustrating its potent stabilizing properties toward the protein. Consistent with the above, all complexes have peaks of RMSF probability distribution that are ~0.1 nm away from each other in the complexes compared to free WEE2 (Figure 3D). Thus, all these analyses supported the equilibration and structural stability of WEE2 in the ligand-bound states through full-length RMSD and per-residue RMSF, suggesting that Midostaurin and Nilotinib increased the stability of the conformation of WEE2 and reinforced the feasibility of WEE2 as a drug target.

#### 3.4.2. Structural Compactness

**Figure 4:**
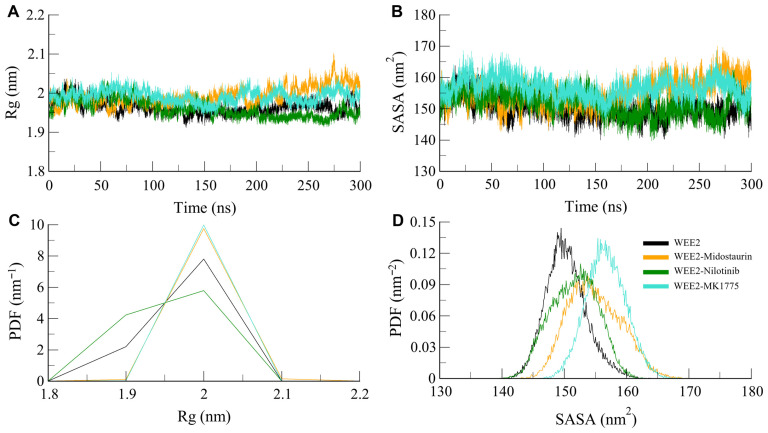
Compactness and solvent exposure of WEE2 during simulations. (**A**) Time evolution of the radius of gyration (*R*_g_). (**B**) Solvent-accessible surface area (SASA). (**C**,**D**) The lower panels display PDFs of *R*_g_ and SASA distributions.

The radius of gyration (*R*_g_) is an important statistical parameter for measuring the folding feature of proteins as well as the global compactness of proteins in protein–ligand complexes [52]. We derived the time-dependent *R*_g_ from the simulation trajectories of WEE2 and its complexes with both Midostaurin, Nilotinib, as well as MK1775. The average *R*_g_ values were 1.96 nm for WEE2, 1.99 nm for WEE2–Midostaurin, 1.96 nm for WEE2–Nilotinib, and 1.96 nm for WEE2-MK1775. Rg profiles showed minimal variation across systems, indicating that ligand binding caused no major alterations in WEE2 packing or folding (Figure 4A). Likewise, Cα-Cα distance distributions remained centered around ~2.0 nm, reflecting stable structural properties throughout the simulations. This conclusion was also supported by probability density analysis, which demonstrated that the probability density of all indicated complexes was greater at 2 nm (Figure 4C). Overall, these results suggest that ligand binding preserves the global compactness of WEE2, with only subtle local adjustments that are consistent with stable and well-maintained protein–ligand conformations.

To further evaluate structural compactness, we calculated solvent-accessible surface area (SASA). Small changes in SASA were observed upon ligand binding, indicating modest burial of surface-exposed regions. These variations remained stable during the trajectory, reflecting preservation of the overall protein fold. WEE2, WEE2–Midostaurin, WEE2–Nilotinib, and WEE2-MK1775 had average SASA values of 150.30 nm^2^, 154.89 nm^2^, 151.57 nm^2^, and 156.70 nm^2^, respectively. The combined effect yielded a slight SASA change upon ligand binding, indicating a partial burial of surface residues that reflect a flexible structure (Figure 4B). The simulation analysis revealed SASA values remained relatively constant during the simulation, further emphasizing WEE2’s general structural stability. Analysis of the SASA indicated that distributions of SASA were centered at ~150 nm^2^ for all systems (Figure 4D). In conclusion, *R*_g_ and SASA analysis showed that the binding of Midostaurin and Nilotinib increased the compactness of WEE2, and it retained its structured state. These results validate the complex stability of WEE2–ligand complexes and provide structural information that may be useful when designing potential non-hormonal contraceptive and infertility modulators.

#### 3.4.3. Dynamics of Interactions in the WEE2 Complexes

**Figure 5:**
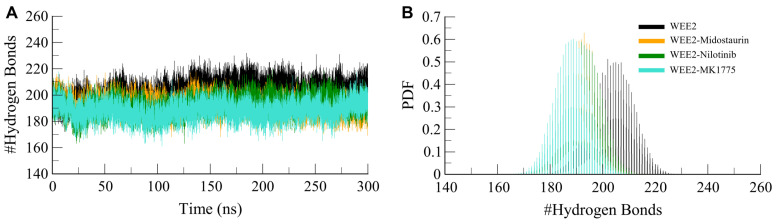
Intramolecular hydrogen bond analysis of WEE2 systems. (**A**) Average number of hydrogen bonds in free and ligand-bound states. (**B**) Probability distribution of intramolecular hydrogen bond counts during simulations.

Intramolecular hydrogen bonds, formed between different regions of a polypeptide chain, play a key role in stabilizing the secondary and tertiary structure of proteins [53]. To determine the effect of ligand binding on WEE2 stabilization, we calculated the intramolecular hydrogen bond profiles of WEE2-free, WEE2–Midostaurin, WEE2–Nilotinib, and WEE2-MK1775 during the simulations (Figure 5). The average hydrogen bonds in WEE2-free, WEE2–Midostaurin, WEE2–Nilotinib, and WEE2-MK1775 were determined as 204, 193, 193, and 188, respectively (Figure 5A). Upon binding of Midostaurin and Nilotinib, a small decrease in the intramolecular hydrogen bonds was recorded compared to the free form of WEE2. The implication of this is that ligands have occupied the intramolecular space within the protein-binding pocket, making it more rigid. Interestingly, the WEE2–Midostaurin and WEE2–Nilotinib complexes displayed the same number of hydrogen bonds, as indicated by the PDF analysis, where the distributions of intramolecular hydrogen bonds in the ligand-bound complexes were shifted towards lower values relative to free WEE2 (Figure 5B). This supports a stronger tendency for WEE2 to retain stable intramolecular hydrogen bond networks in the presence of Midostaurin or Nilotinib. These observations together suggest that ligand binding not only decreases conformational fluctuations and compactness but also preserves the intramolecular hydrogen bonding networks in WEE2 with minor fluctuations.

#### 3.4.4. Secondary Structure Dynamics

**Figure 6:**
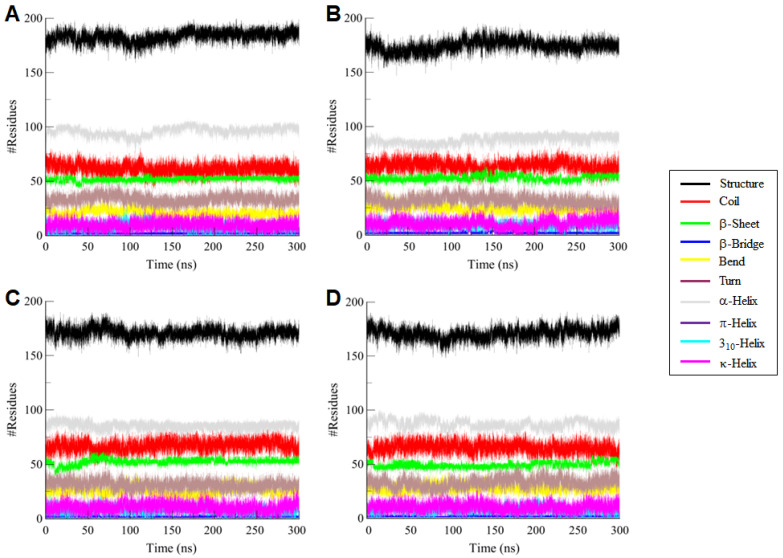
Time-resolved secondary structure patterns of (**A**) free WEE2, (**B**) WEE2–Midostaurin, (**C**) WEE2–Nilotinib, and (**D**) WEE2-MK1775 monitored throughout the 300 ns simulation, illustrating the structural stability and conformational transitions upon ligand binding.

To assess the structural impact of ligand binding on WEE2, we analyzed the distribution of secondary structure elements over a 300 ns MD simulation. Variations in α-helices, β-sheets, turns, and coils can reflect conformational flexibility or ligand-induced structural rearrangements. Figure 6 illustrates the time-dependent secondary structure profiles of unbound WEE2 and its complexes with Midostaurin, Nilotinib, and MK1775, while the average structural composition is summarized in Table 4. WEE2 maintained a largely stable secondary structure throughout the trajectory (Figure 6A). The WEE2–Midostaurin complex displayed an increase in coil and a decrease in helix content, accompanied by a slight reduction in turn residues, suggesting enhanced flexibility (Figure 6B). In the WEE2–Nilotinib complex, α-helix content decreased modestly while most structural features remained stable with a minor increase in sheet formation (Figure 6C). The WEE2-MK1775 complex showed negligible deviations in the structural features, where sheets and helix formation had decreased (Figure 6D). Overall, these subtle changes indicate that ligand binding induces only minor conformational adjustments in WEE2, without compromising its structural stability or functional integrity.

### 3.5. Principal Component Analysis

**Figure 7:**
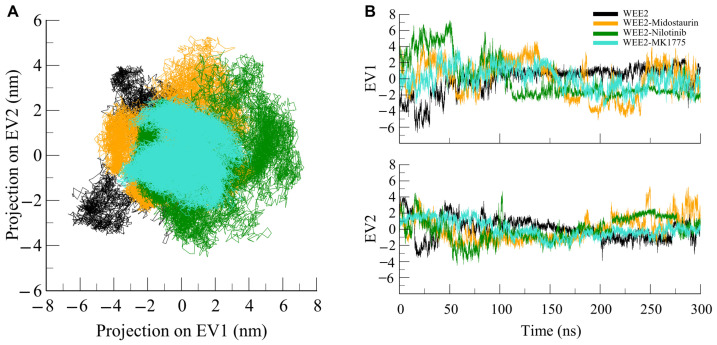
Principal component analysis (PCA) of WEE2 dynamics. (**A**) Two-dimensional projection of PCA trajectories along the first two principal components (PC1 and PC2) for WEE2 and its complexes with Midostaurin, Nilotinib, and MK1775. (**B**) Time evolution of PCA trajectories along the first two eigenvectors (EV1 and EV2) for WEE2 and its complexes with Midostaurin, Nilotinib, and MK1775.

PCA is a well-established method for investigating global motions of proteins and their conformational space during MD simulations [54]. PCA projects the simulation trajectories onto a few dimensions and reveals the most sensitive motions that govern the flexibility and stability of protein–ligand complexes. We evaluated the first two principal components (PC1 and PC2) that show maximum variances in atomic displacements to investigate conformational dynamics of WEE2 and its complexes with Midostaurin, Nilotinib, and MK1775. Conformational sampling of the WEE2 systems in the essential subspace (projected onto the eigenvectors EV1 and EV2 from the *C*α atoms) is shown in Figure 7. The free WEE2 adopted a more extensive conformational space, suggesting greater flexibility in the absence of ligands. In striking contrast, the conformational sampling of WEE2 became markedly restricted upon bound ligand, which strongly suggests that the protein-bound ligands constrain the set of conformations available to the protein and thus increase stability (Figure 7A). Within the ligand-bound complexes, both the WEE2–Midostaurin and WEE2–Nilotinib complexes showed similar phase spaces compared to the free protein, and the reference complex showed the tightest distributions along both eigenvectors (Figure 7B). Conversely, the WEE2–Nilotinib complex showed wider projections along both eigenvectors compared to Midostaurin and MK1775, indicating greater conformational flexibility and less stability. In summary, PCA showed that ligand binding does not fundamentally change the global fold of WEE2 but affects how far it samples different conformations. Midostaurin and MK1775 promote a tighter and more stable conformational landscape, while Nilotinib permits larger ensemble oscillations.

### 3.6. Free-Energy Landscape Analysis

**Figure 8:**
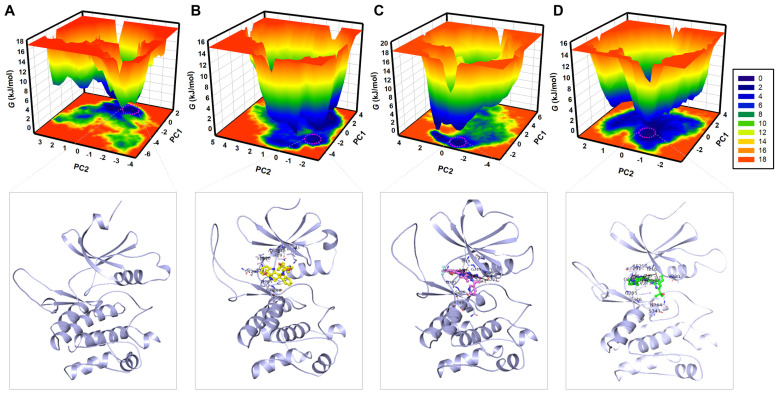
Free-energy landscape (FEL) of WEE2 systems. Contour maps show conformational basins and global minima for (**A**) free WEE2, (**B**) WEE2–Midostaurin, (**C**) WEE2–Nilotinib, and (**D**) WEE2-MK1775. The darker blue regions indicate the most energetically favorable conformations, reflecting the thermodynamic stability of WEE2–ligand interactions. The lower panels display the corresponding protein structural snapshot extracted from the global minimum.

FEL analysis is a powerful tool that allows us to characterize the conformational dynamics and folding states of proteins by providing information about the basins’ free-energy stabilities and the transitions between conformational states during the simulations [44]. To reveal the folding dynamics and conformational stability of WEE2 and its ligand-bound complexes with Midostaurin, Nilotinib, and MK1775, FELs from the PCA trajectory were generated (Figure 8). The FEL for WEE2 showed a single global minimum confined within 1–2 primary basins, with the deepest minima indicated with the darkest blue (Figure 8A). The shape of this distribution implies that the protein is flexible and able to exist in multiple energetically favorable conformations over the course of the simulation. FELs showed that significant differences were found after ligand binding. Compared to the free protein, the WEE2–Midostaurin complex sampled multiple low-energy conformations within a broader (but still well-defined) energy basin (Figure 8B). This reflects the presence of several energetically accessible microstates rather than instability. This interpretation aligns with RMSF and PCA results, which indicate that Midostaurin stabilizes local fluctuations while permitting exploration of nearby substates. At the same time, the FEL of the WEE2–Nilotinib complex demonstrated tighter and more focused energy basins, along with an observed tight global minimum (Figure 8C). The presence of a global minimum indicates that the conformation is stable, and the reduced conformational diversity compared to Midostaurin may suggest that WEE2 dynamics are less effectively stabilized. This observation is representative of a stable folding state and limited conformational transitions, indicating that this complex has greater stability. Similarly to the WEE2–Midostaurin complex, the FEL for the WEE2-MK1775 complex displayed a wide landscape featuring multiple global minima predominantly located within 2–3 basins (Figure 8D).

Overall, FEL analysis outlined the propensity of WEE2 in terms of both folding behavior and conformational stability for the WEE2 and ligand complexes. The ligand-bound states of WEE2 showed multiple stabilities, but Nilotinib binding led to an extremely compact and stable conformation landscape. These findings highlight the stabilizing influence of Midostaurin and Nilotinib on what would otherwise be WEE2 dynamic behavior and provide compelling evidence for their further pursuit as novel non-hormonal contraceptive agents and possible regulators of fertility. The FEL analysis revealed distinct conformational preferences of WEE2 in its free and ligand-bound states (Figure 8, lower panels). Structural snapshots extracted from the global minima illustrate the most stable conformations adopted in each system, providing mechanistic insight into how Midostaurin, Nilotinib, and MK1775 differentially influence the stability and flexibility of WEE2. Notably, all three compounds remained stably bound within the ATP-binding pocket throughout the simulations, reinforcing their potential as effective WEE2 binders.

### 3.7. MM-PBSA Analysis

MM-PBSA calculations were conducted to assess the calculated binding affinities between the selected ligands and WEE2 based on snapshots extracted from the production-phase MD trajectories. The obtained Δ*G*_binding_ was broken down into components representing the energetic contributions due to various interactions, such as the gas-phase components (van der Waals and electrostatic interactions) and polar and non-polar solvation contributions (Table 5). The Δ*G*_binding_ values for Midostaurin and Nilotinib were also more favorable than those for MK1775, indicating more robust and stable interactions of Midostaurin and Nilotinib with WEE2. More specifically, values of –18.78 ± 2.23 kJ/mol (WEE2–Midostaurin complex) and –17.47 ± 2.95 kJ/mol (WEE2–Nilotinib complex) were found for the binding free energy. In comparison, the WEE2-MK1775 complex was slightly less stable, presenting a Δ*G*_binding_ value of –15.79 ± 3.92 kJ/mol. All three ligands form stable and energetically favorable complexes with WEE2, as indicated by consistently negative Δ*G*_binding_ values. Midostaurin and Nilotinib demonstrated relatively higher binding affinities than the other compounds, and this was consistent with the results of the molecular docking study. In summary, MM-PBSA analysis highlights Midostaurin and Nilotinib as attractive candidates for repurposing as WEE2-targeting agents, providing a strong rationale for further biochemical and cellular validation. We recommend that these hits be subjected to ML/DL rescoring as an efficient next step to refine prioritization for in vitro testing and to estimate potential off-target or toxicity concerns.

### 3.8. Limitations and Remarks

A major limitation of this study is the absence of experimental validation. All conclusions are based on computational analyses, and thus, the predicted inhibitory activity of Midostaurin and Nilotinib must be interpreted cautiously. We recommend biochemical kinase assays, WEE2–WEE1 selectivity profiling, oocyte maturation studies, and ATP-competition assays as essential next steps to confirm our computational predictions. The compounds Midostaurin and Nilotinib emerged as computational hits due to strong WEE2 binding features, not because they are suitable contraceptive agents in their current form. We emphasize that this study does not propose clinical contraceptive use of cytotoxic drugs. Instead, the findings should be interpreted as a demonstration of how in silico screening can uncover structural scaffolds with WEE2-binding potential, which may guide the future design of safer and more selective inhibitors. A literature survey confirmed that Midostaurin and Nilotinib have no reported contraceptive or fertility-suppressing side effects in clinical studies. Their adverse effects primarily stem from multi-kinase inhibition unrelated to reproductive pathways. This further supports our reframed interpretation that these drugs serve as structural starting points rather than contraceptive candidates.

## 4. Conclusions

In this study, we used an integrated structure-based screening and molecular dynamics workflow to identify repurposed small-molecule scaffolds with the potential to bind and stabilize the WEE2 catalytic domain. Using this integrated approach, two clinically approved compounds, Midostaurin and Nilotinib, with favorable drug-likeness, relevant predicted biological activities, and relatively high binding affinities toward WEE2 were identified. Structural dynamics and conformational analyses, including RMSD, RMSF, *R_g_*, SASA, hydrogen-bond characterization, PCA, and FEL mapping, showed that both ligands bind to WEE2 and induce only minimal conformational perturbations during the simulations. MM-PBSA calculations strengthened their binding stability and energetics over MK1775. This study demonstrates a computational screening pipeline for identifying FDA-approved molecules with structural compatibility for WEE2 binding. Rather than proposing immediate contraceptive drug candidates, our results illustrate how integrated docking, MD simulations, and ML-informed prioritization can highlight scaffold classes with potential relevance for future WEE2-targeted drug design. However, the results are predictive, as they are based solely on computational simulations. Finally, the study suggest that integrating ML- and DL-based rescoring and ligand property predictors into structure-based pipelines offers a rapid, cost-effective approach to prioritize repurposed drug candidates before further experimental testing. Adopting such ML/DL steps for the top candidates identified here would increase confidence in selecting leads for biochemical validation and accelerate translational progress. Subsequently, future work should therefore focus on experimental validation, including biochemical binding assays, oocyte maturation studies, and animal models.

## Figures and Tables

**Table 1:** Docking and binding energy parameters of the top-ranked compounds against WEE2 compared with the reference inhibitor MK1775.

S. No.	Drug	Binding Energy (kcal/mol)	pKi	Ligand Efficiency (kcal/mol/Non-H Atom)	Torsional Energy
1.	Temoporfin	−12.6	9.24	0.2423	2.4904
2.	Dutasteride	−12.1	8.87	0.327	1.2452
3.	Midostaurin	−11.5	8.43	0.2674	1.8678
4.	Rifaximin	−11.5	8.43	0.2018	2.1791
5.	Ergotamine	−11.4	8.36	0.2651	1.5565
6.	Nilotinib	−11.3	8.29	0.2897	2.1791
7.	Radotinib	−11.1	8.14	0.2846	2.1791
8.	Pazopanib	−10.7	7.85	0.3452	1.8678
9.	Fentonium	−10.7	7.85	0.2972	3.113
10.	Ponatinib	−10.6	7.77	0.2718	2.1791
11.	MK1775 (Adavosertib)	−8.5	6.23	0.25	2.1791

**Table 2:** Pharmacological profiles of the selected WEE2 hits. The table presents the primary targets, approved therapeutic uses, and relevance to kinase biology and fertility modulation for the 10 shortlisted FDA-approved drugs. This profiling was used to identify Midostaurin and Nilotinib as the most promising candidates for downstream analyses.

S. No.	Drug	Known Target(s)	Approved Therapeutic Use(s)	Relevance to WEE2/Contraception Context
1.	Temoporfin	Photosensitizer (ROS generation upon light activation)	Photodynamic therapy for cancers	Not relevant to kinase inhibition; unlikely candidate
2.	Dutasteride	5α-reductase (Type I and II) inhibitor	Benign prostatic hyperplasia, androgen-related disorders	Targets reproductive hormone metabolism; relevant for fertility modulation
3.	Midostaurin	FLT3, KIT, PDGFR, VEGFR2, PKC (multi-kinase inhibitor)	Acute myeloid leukemia, systemic mastocytosis	Strong kinase inhibitory profile; mechanistically relevant to WEE2 inhibition
4.	Rifaximin	Bacterial RNA polymerase	Traveler’s diarrhea, hepatic encephalopathy, irritable bowel syndrome (IBS-D)	Antibacterial, no relevance to fertility or kinase targeting
5.	Ergotamine	5-HT receptors, adrenergic receptors	Migraine treatment	Neurovascular drug, not directly relevant
6.	Nilotinib	BCR-ABL, c-KIT, PDGFR	Chronic myeloid leukemia	Potent kinase inhibitor; mechanistically relevant to WEE2 inhibition
7.	Radotinib	BCR-ABL	Chronic myelogenous leukemia (CML) (approved in Korea)	Similarly to nilotinib, a kinase inhibitor, a possible candidate
8.	Pazopanib	VEGFR, PDGFR, c-KIT	Renal cell carcinoma, soft tissue sarcoma	Kinase inhibitor, but primarily angiogenesis-related; limited fertility relevance
9.	Fentonium	Muscarinic receptor antagonist	Antispasmodic agent	No relevance to kinase or fertility
10.	Ponatinib	BCR-ABL (incl. T315I mutant), VEGFR, FGFR, KIT	Resistant CML, Philadelphia chromosome-positive Acute Lymphoblastic Leukemia (Ph+ ALL)	Kinase inhibitor, but with severe cardiovascular toxicity; not ideal for contraceptive application
11.	MK1775 (Adavosertib)	WEE1 kinase inhibitor	Cancer (clinical trials)	Reference compound validates the approach for WEE family

**Table 3:** Molecular dynamics parameters of WEE2 and its complexes with Midostaurin, Nilotinib, and MK1775. Average values for RMSD, RMSF, *R*_g_, SASA, and hydrogen bond counts are presented. ‘#H-Bonds’ represents the intramolecular hydrogen bonds within WEE2.

Protein/Protein–Ligand Complex	RMSD (nm)	RMSF (nm)	*R_g_* (nm)	SASA (nm^2^)	#H-Bonds
WEE2	0.31	0.13	1.96	150.30	204
WEE2–Midostaurin	0.34	0.16	1.99	154.89	193
WEE2–Nilotinib	0.34	0.15	1.96	151.57	193
WEE2-MK1775	0.24	0.13	1.99	156.70	188

**Table 4:** Average distribution of secondary structure elements in WEE2 and its ligand-bound complexes during a 300 ns molecular dynamics simulation. The analysis reports the mean number of residues adopting each structural category, coil, β-sheet, β-bridge, bend, turn, α-helix, 3_10_-helix, and π-helix, across all trajectory frames. This evaluation reveals how ligand binding influences the structural stability and dynamic flexibility of WEE2.

Element	WEE2	WEE2–Midostaurin	WEE2–Nilotinib	WEE2-MK1775
Coil	61	64	67	65
β-sheet	52	52	53	49
β-bridge	2	2	2	2
Bend	21	25	26	28
Turn	33	32	31	33
α-helix	96	88	86	87
π-helix	0	0	0	0
3_10_-helix	8	9	7	8
κ-Helix	10	11	11	11

**Table 5:** MM-PBSA binding free-energy estimates for WEE2–ligand complexes. Energetic contributions of van der Waals, electrostatic, polar solvation, and non-polar solvation terms are shown for Midostaurin, Nilotinib, and MK1775.

Complex	Δ*E*_vdW_	Δ*E*_EL_	Δ*E*_PB_	Δ*E*_NPOLAR_	Δ*G*_GAS_	Δ*G*_SOLV_	∆$G_{Total}\;(kJ/mol)$
WEE2–Midostaurin	−43.37	−5.25	34.37	−4.54	−48.61	29.83	−18.78 ± 2.23
WEE2–Nilotinib	−37.23	−17.62	41.44	−4.07	−54.84	37.37	−17.47 ± 2.95
WEE2-MK1775	−36.08	−7.82	32.19	−4.09	−43.89	28.10	−15.79 ± 3.92

## Data Availability

All data generated or analyzed during this study are included in this article.

## Supplementary Materials

The following supporting information can be downloaded at: https://www.mdpi.com/article/10.3390/life16020185/s1, Figure S1: WEE2 bound to co-crystallized MK1775 (yellow) and the docked MK1775 pose (green). The figure was generated in PyMOL using PDB structure 5VDK.
