# Supplementary material for: Identification of FDA-Approved Drugs as Potential Inhibitors of WEE2: Structure-Based Virtual Screening and Molecular Dynamics with Perspectives for Machine Learning-Assisted Prioritization
Source: Life (Basel). 2026 Jan 23;16(2):185. doi: 10.3390/life16020185 (PMC12941930; doi:10.3390/life16020185)
Supplement: Supplementary file 1 [file life-16-00185-s001.zip › life-4099659-supplementary.PPTX]

## Slide 1
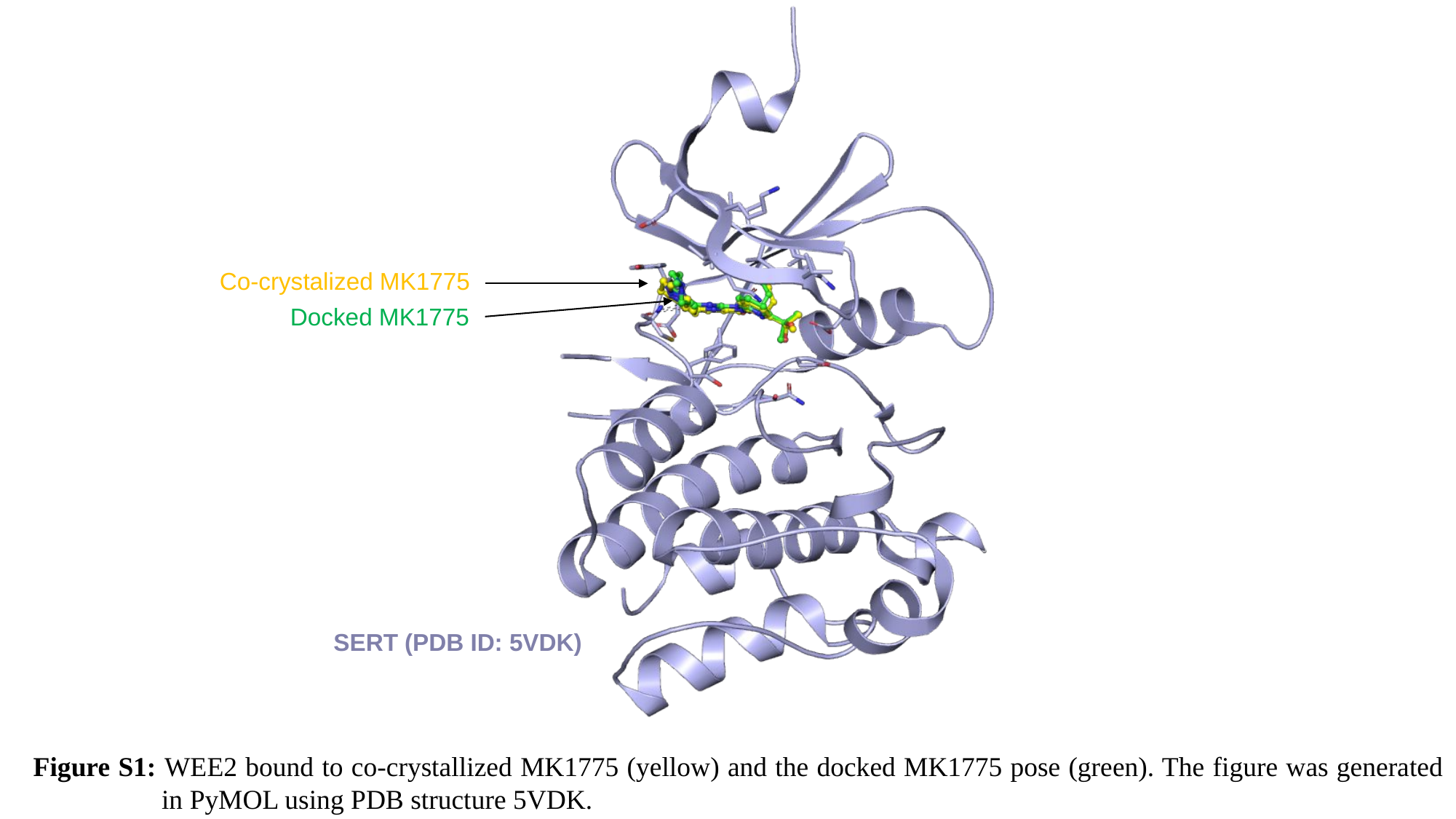

Co-crystalized MK1775
Docked MK1775
SERT (PDB ID: 5VDK)
Figure S1: WEE2 bound to co-crystallized MK1775 (yellow) and the docked MK1775 pose (green). The figure was generated in PyMOL using PDB structure 5VDK.
